# Combined linkage and association mapping reveal QTL for host plant resistance to common rust (*Puccinia sorghi*) in tropical maize

**DOI:** 10.1186/s12870-018-1520-1

**Published:** 2018-11-29

**Authors:** Hongjian Zheng, Jiafa Chen, Chunhua Mu, Dan Makumbi, Yunbi Xu, George Mahuku

**Affiliations:** 1 CIMMYT-China Specialty Maize Research Center, Shanghai Academy of Agricultural Sciences, Shanghai, China; 2 Crop Breeding and Cultivation Research Institute, Shanghai Academy of Agricultural Sciences, Shanghai, China; 3 International Maize and Wheat Improvement Center (CIMMYT), Apdo, Postal 6-641, 06600 Mexico, DF Mexico; 4 College of Life Sciences, Synergetic Innovation Center of Henan Grain Crops and National Key Laboratory of Wheat and Maize Crop Science, Henan Agricultural University, Zhengzhou, 450002 China; 5 Maize Research Institute, Shandong Agricultural Academy of Sciences, Jinan, China; 6 International Maize and Wheat Improvement Center (CIMMYT), P.O Box 1041-00621, Nairobi, Kenya; 7 Institute of Crop Sciences, Chinese Academy of Agricultural Sciences, Beijing, China; 8 International Institute of Tropical Agriculture (IITA), P.O. Box, 34443, Dar es Salaam, Tanzania

**Keywords:** *Puccinia sorghi*, Genome-wide association study, maize diseases, Single-nucleotide polymorphisms

## Abstract

**Background:**

Common rust, caused by *Puccinia sorghi*, is an important foliar disease of maize that has been associated with up to 50% grain yield loss. Development of resistant maize germplasm is the ideal strategy to combat *P. sorghi*.

**Results:**

Association mapping performed using a mixed linear model (MLM), integrating population structure and family relatedness identified 25 QTL (*P* < 3.12 × 10^− 5^) that were associated with resistance to common rust and distributed on chromosomes 1, 3, 5, 6, 8, and 10. We identified three QTLs associated with all three disease parameters (final disease rating, mean disease rating, and area under disease progress curve) located on chromosomes 1, 3, and 8. A total of 5 QTLs for resistance to common rust were identified in the RIL population. Nine candidate genes located on chromosomes 1, 5, 6, 8, and 10 for resistance to common rust associated loci were identified through detailed annotation.

**Conclusions:**

Using a diverse set of inbred lines genotyped with high density markers and evaluated for common rust resistance in multiple environments, it was possible to identify QTL significantly associated with resistance to common rust and several candidate genes. The results point to the need for fine mapping common rust resistance by targeting regions identified in common between this study and others using diverse germplasm.

**Electronic supplementary material:**

The online version of this article (10.1186/s12870-018-1520-1) contains supplementary material, which is available to authorized users.

## Background

Common rust of maize, caused by *Puccinia sorghi* Schwein, is widely distributed in tropical, subtropical, temperate, and highland environments, where it causes economic losses on approximately 7.8 million ha or 34% of the maize area [1]. Substantial losses in forage quality and up to 50% loss in grain yield have been observed [2]. Damage is caused by loss of photosynthetic leaf area, chlorosis and premature leaf senescence, leading to incomplete grain filling and poor yields. Common rust can be controlled by use of fungicides or resistant cultivars. For economic and ecological reasons, development and deployment of resistant maize cultivars is the most appropriate strategy to minimize the effects of *P. sorghi*, and significantly contribute to increased grain yield [3].

Previous research revealed that resistance of maize to common rust is controlled by both quantitative and qualitative genes [4–8]. Qualitative or major-gene resistance is controlled by single major-effect resistance genes that are either dominant or recessive and generally provide race-specific, high-level resistance, but in a non-durable manner. In contrast, quantitative resistance typically has a multi-genic basis and generally provides non-race-specific intermediate levels of resistance. In maize, more than 25 dominant *Rp* genes are involved in race-specific resistance for common rust and are organized in complex loci at chromosomes 3, 4, 6 and 10 [3, 9, 10]. Fourteen different resistance genes have been designated as *Rp1-A* to *Rp1-N* based on map position [11, 12] and a number of these have been genetically recombined, suggesting that they are encoded by members of a gene cluster [12, 13]. Subsequently, other genes from the *rp1 loci* designated *rp5* and *rp6* on chromosome 10 [12, 14] *rp*3 and *rp*4 on chromosomes 3 and 4, respectively [15], *Rp7* [16] and *Rp8 on chromosome 6* [5] have been reported. The *Rp1-D* gene on chromosome 10 was cloned from the *HRp1-D* haplotype using transposon tagging [17], and further validated via a complementation test [18]. The *Rp1* cluster was shown to vary widely in copy number (1–52 copies) among different maize haplotypes [19].

Single race-specific or major resistance genes confer high levels of resistance to specific rust biotypes, but simply inherited resistance may result in selection for virulent races. Although it is easier to work with qualitative resistance in crop genetic research and breeding, partial resistance to the diseases may be more durable than simply inherited resistance [20–22]. However, partial resistance has been more difficult to transfer than simply inherited resistance due to its presumed multigenic nature. Molecular mapping techniques in combination with marker-assisted selection, however, may enable breeders to more effectively identify and exploit this type of resistance.

Since the first mapping study of quantitative trait loci (QTL) in a plant was published in 1986 [23] a substantial number of studies have been conducted to map QTL for different disease resistances [3, 6, 7, 24–26]. Lübberstedt et al. [3] used European maize flint lines and identified 20 QTL conferring partial resistance to common rust distributed over all 10 maize chromosomes. Kerns et al. [6] used a segregating population from cross FRMo17 × BS11 (FR)c7 and identified 24 molecular markers in 16 chromosomal regions that were significantly associated with partial rust resistance. Brown et al. [24], using an F_2:3_ population from a cross between sweet corn inbred lines IL731a and W6786, identified nine regions on six chromosomes, which were significantly associated with common rust severity. These mapping studies thus far have provided information on the genetic architecture of resistance to common rust, including the number, location, and action of chromosomal segments. Through linkage mapping, several *P. sorghi* resistance QTL have been identified [3–6, 8, 24], but these have not been validated for utilization by breeders. It is, therefore, important to identify new genes for resistance to common rust that can be effectively used in tropical maize breeding programs.

Genome-wide association studies (GWAS), based on linkage disequilibrium (LD) analysis, have become a useful tool for identifying and mapping causal genes with modest effects like common rust resistance genes [27, 28]. Three loci (chromosome 2, chromosome 3 and chromosome 8) associated with maize common rust resistance in temperate maize germplasm were identified using GWAS [8]. GWAS is particularly useful when large numbers of inbred lines are available, because once these lines have been genotyped they can be phenotyped in different environments across seasons/years, making it possible and cost-effective to study the genetic architecture of different traits using phenotypic data from multiple environments [28, 29]. The traditional QTL mapping in bi-parental populations is powerful in comparing pairs of alleles, which gives a lower false discovery rate compared to GWAS. Hence, combining both GWAS and traditional QTL mapping maybe a powerful method for discovering causal loci across the genome [26, 30]. In this study, we used GWAS in a diverse panel of tropical maize inbred lines and QTL mapping in a recombinant inbred line (RIL) population to analyze chromosomal regions associated with resistance to *P. sorghi.* The objectives were to localize and estimate the effects of minor and major loci for resistance to common rust using high density single nucleotide polymorphism (SNP) markers, and to identify candidate genes and potential causal polymorphisms for resistance to common rust through detailed annotation.

## Results

### Phenotypic diversity

The GWAS panel was evaluated at six environments for response to common rust and ratings were done three times for all environments except at Kenya09, where lines were evaluated once. Results showed very strong significant correlation between the three disease traits (AUDPC, FDR and MDR) (Table 1). Because disease rating at Kenya09 was evaluated once and strong correlation was observed between the three disease parameters, further analysis was conducted using only the FDR data. A weak negative correlation was observed between maturity (AD and SD) and rust resistance parameters (Table 1). Although rust resistance is a complex trait, the inoculum pressure was consistently high under field conditions and we obtained highly reliable phenotypic data, as shown by the within location repeatability of FDR that was ≥0.76 (Table 2). The histogram of FDR at each of the six environments showed a continuous distribution (Additional file 1), which suggested quantitative resistance genes might be responsible for most of the variation.

**Table 1:** Pearson correlation coefficients between three disease parameters and flowering traits

	AUDPC	FDR	MDR	AD	SD
AUDPC	1	.	.	.	.
FDR	0.97**	1	.	.	.
MDR	0.98**	0.98**	1	.	.
AD	−0.25	−0.25	− 0.24	1.00	.
SD	−0.16	−0.12	− 0.12	0.99**	1.00

*AUDPC* Area under the disease progress curve, *FDR* Final disease rating, *MDR* Mean disease rating, *AD* Days to anthesis, *SD* Silking date

**indicates significant at *P* < 0.01

**Table 2:** Summary statistics and repeatability for final disease rating of common rust in a set of 296 DTMA panel inbred lines in six environments

	Location					
Statistics	BM09A	BM09B	Kenya09	BM10	BM11	Celaya12
Range	1.0–4.5	1.0–4.5	1.0–5.0	1.0–5.0	1.0–4.0	1.0–5.0
Mean (Panel)	1.81	1.98	2.31	2.79	1.75	2.52
Mean (Resistant checks)	1.5	1.92	–	1	1.17	1.33
Mean (Susceptible checks)	3.75	4.17	–	4.67	4.25	4.77
Repeatability	0.88	0.76	0.88	0.92	0.78	0.95
LSD_(0.05)_^a^	0.89	0.98	0.79	0.66	0.72	0.51
CV (%)^b^	49.4	39.9	33.8	41.3	31.5	33.3

^a^*LSD* Least significant difference

^b^*CV* coefficient of variation

**Fig. 1:**
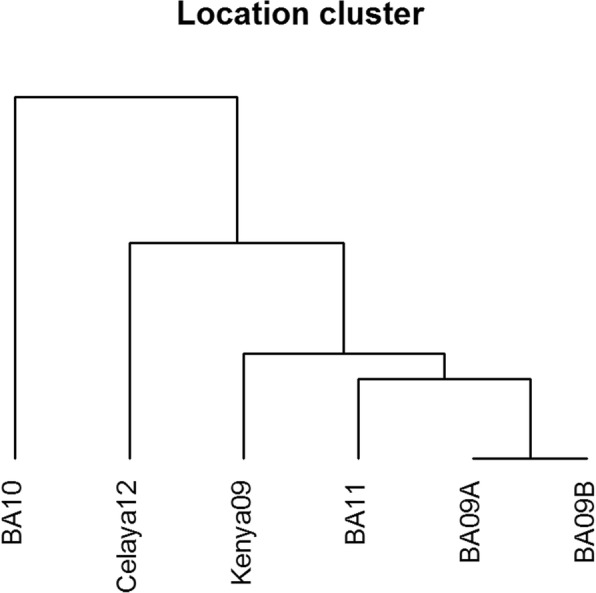
Dendrogram of six environments used to evaluate the Drought Tolerant Maize for Africa (DTMA) panel of 296 inbred lines for reaction to common rust. The Ward minimum variance method was used to group environments based on genetic correlations

Highly significant differences (*P* < 0.001) among lines, environments and line × environment interaction were observed for FDR of common rust in the DTMA panel of inbred lines (Table 3). Several inbred lines exhibited differential response to common rust in various environments (Additional file 2). Genetic correlations for FDR among locations ranged from 0.48 to 1.00 (Table 4). Despite the significant line × environment interactions, strong genetic correlation coefficients among most of the environments were observed for FDR scores. Clustering of environments using FDR revealed two major clusters, with BA10 separated from other environments (Fig. 1). Environment BA10 had the smallest genetic correlations with other environments and was excluded from further analysis. The year of common rust evaluation at this location (2010) was extremely dry and therefore disease expression was affected.

**Table 3:** Combined analysis of variance for final disease rating of common rust in a set of 296 Drought Tolerant Maize for Africa panel of inbred lines using combined data from evaluations conducted in 2009 to 2012

Source of variation	Degrees of freedom	Sum of squares	Mean square	*F* value	*P* value
Environment (Env)	5	800.79	160.16	731.31	< 1.0E-10
Line	295	2281.83	7.74	35.32	< 1.0E-10
Replication (Rep)/Env	12	11.59	0.97	4.41	4.99E-07
Env × Line	1450	1461.41	1.01	4.60	< 1.0E-10
Block(Env × Rep)	687	217.12	0.32	1.44	< 1.0E-10
Error	2782	609.26	0.22

**Table 4:** Genetic (upper diagonal) and phenotypic correlations (below diagonal) for final disease rating (FDR) of common rust among locations

	BA09A	BA09B	Kenya09	BA10	BA11	Celaya12
BA09A	1	1.00	0.86	0.55	0.86	0.69
BA09B	0.79	1	0.88	0.55	0.92	0.73
Kenya09	0.73	0.69	1	0.62	0.82	0.70
BA10	0.36	0.35	0.42	1	0.52	0.48
BA11	0.61	0.57	0.66	0.39	1	0.77
Celaya12	0.67	0.59	0.74	0.47	0.73	1

All the correlations were significant at the *P* < 0.01 level

### Genetic structure of DTMA panel of inbred lines

**Fig. 2:**
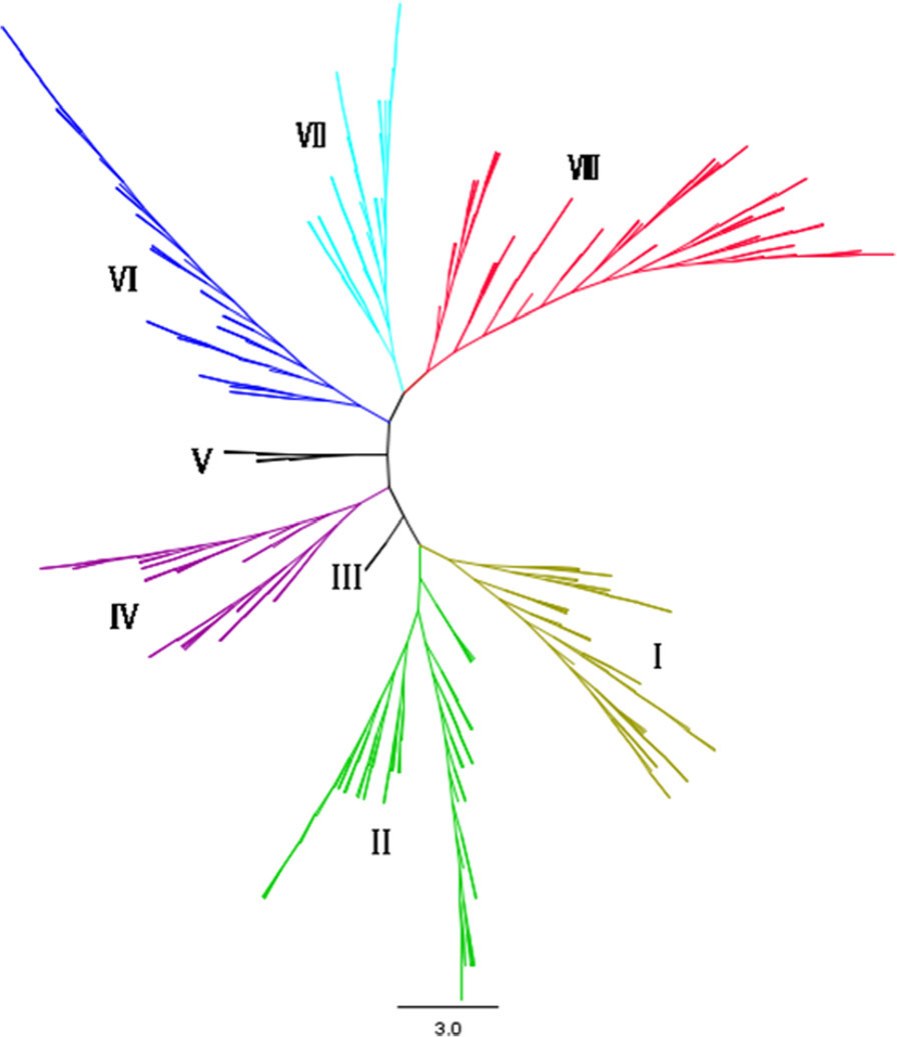
Neighbor-joining tree constructed from a simple matching distance of 32,051 single nucleotide polymorphism (SNP) markers and showing the population structure of the DTMA panel of tropical maize inbred lines. The eight divergent groups identified are color-coded and designated I-VIII

The germplasm collection used in this study included 296 tropical maize inbred lines representing a large amount of the genetic diversity of CIMMYT and IITA’s stress (drought, low nitrogen, acid soils, diseases, and entomology) breeding programs in Mexico, Colombia, Zimbabwe, Nigeria, Ethiopia and other tropical countries. Among the 55,000 SNP markers used to genotype the lines, 39,996 SNPs were scored for all lines. There was an even distribution of minor allele frequency across the 39,996 SNPs, out of which 7945 SNP markers (19.8%) had a minor allele frequency (MAF) below 5% across all tested lines. A total of 32,051 SNPs were used for population structure and association mapping after excluding SNPs with MAF below 5%. The results showed that the panel had eight divergent groups, namely, I, II, III, IV, V, VI, VII and VIII (Fig. 2 and Additional file 3). Thus, structure analysis separated the germplasm clearly into different divergent groups.

### Genome wide SNP association

**Fig. 3:**
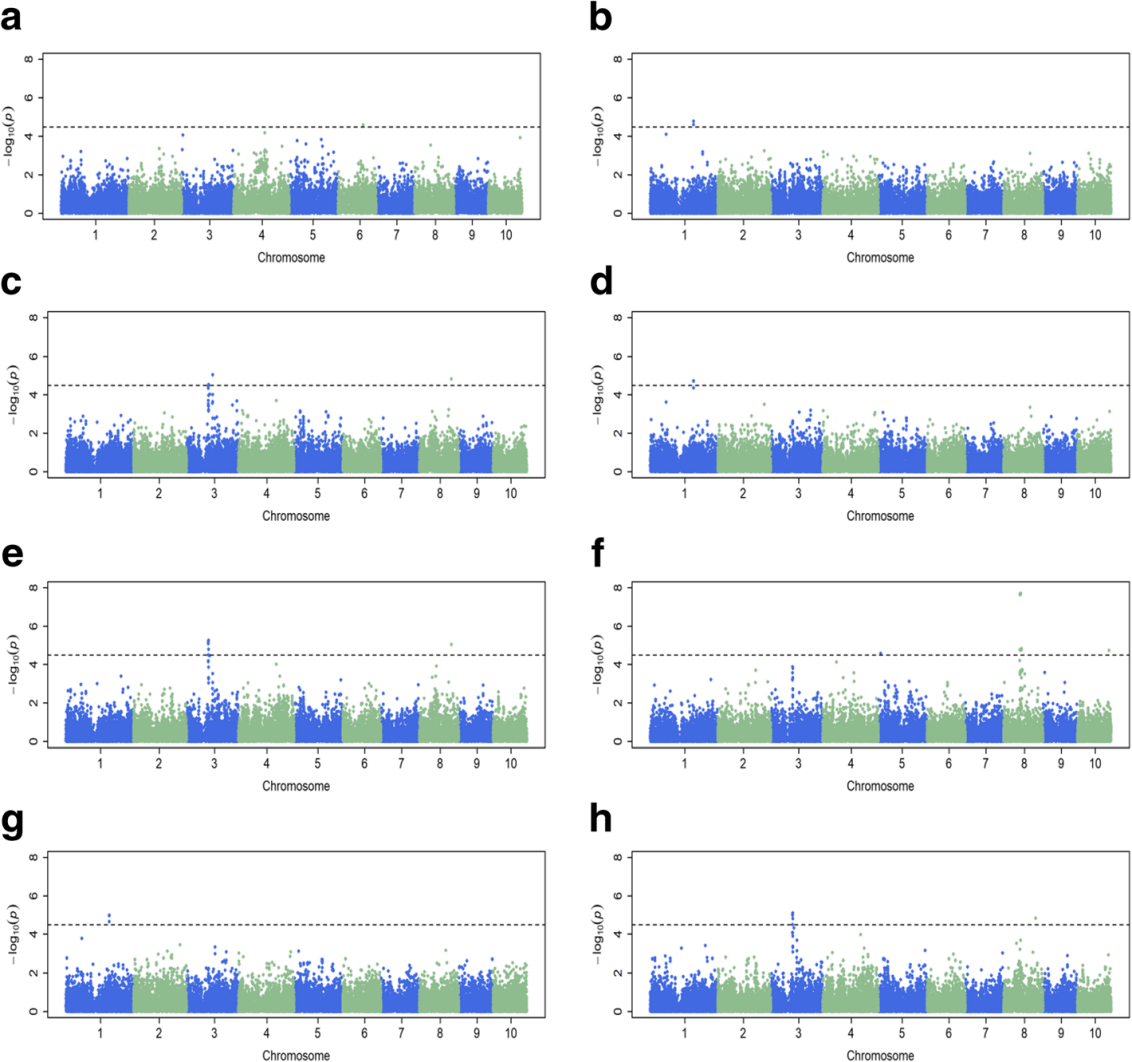
Genome-wide association mapping of common rust resistance with 32,051 SNPs in Drought Tolerant Maize for Africa (DTMA) panel. The vertical axis indicates –log_10_ of *P*-value scores, and the horizontal axis indicates chromosomes and physical positions of SNPs. The dashed lines correspond to the thresholds of Bonferroni correction (*P* < 3 × 10^− 5^). The Manhattan plots for significant SNP marker for different environments and disease evalution parameter. (**a**) One SNP marker on Chr. 6 associated with FDR in EK09; **b**) 3 SNP markers on Chr.1 associated with FDR in BM10; **c**) 3 SNP markers on Chr.3 and Chr.8 associated with FDR in BM11; **d**) 2 SNP markers on Chr.1 associated with MDR in BM10; **e**) 5 SNP markers on Chr.3 and Chr.8 associated with MDR in BM11, respectively; **f**) 14 SNP markers on Chr.5, Chr.8 and Chr.10 associated with AUDPC ted in BM09A; **g**) 3 SNP markers on Chr.1 associated with AUDPC d in BM10; **h**) 6 SNP markers on Chr.3 and 8 associated with AUDPC BM11., respectively

Association mapping was performed using a mixed linear model (MLM) by integrating population structure (PCA) and family relatedness (kinship) within the DTMA panel using 32,051 SNPs with rare alleles (MAF < 5%) having been excluded. A Bonferroni threshold (1/n) was used to show the significant polymorphic SNPs (*P* < 3.12 × 10^− 05^ for 32,051 SNPs). In total, 37 SNP markers associated with common rust resistance were detected. Of the 37 SNP markers, seven SNP markers on four chromosomes (Chrs.1, 3, 6 and 8) were significantly associated with FDR (*P* < 3.12 × 10^− 5^), seven SNP markers on three chromosomes (Chrs.1, 3 and 8) were significantly associated with MDR, and 23 SNP markers on five chromosomes (Chrs.1, 3, 5, 6, 8 and 10) were significantly associated with AUDPC (Table 5, Fig. 3a-h). The percentage of phenotypic variance explained (PVE) by an individual significant SNP ranged from 6.43 to 12.97%. Quantile-quantile plots (QQ plots) showed that population structure was controlled well by the mixed linear model (Additional file 4).

**Table 5:** Association mapping for resistance to common rust of maize in the Drought Tolerant Maize for Africa panel of maize inbred lines

Trait ^a^	QTL	SNP marker^b^	Bin	Pos.	BA09A		BA09B		Kenya09		BA10		BA11		Celaya12	
					*P* ^d^	R^2,e^	*P* ^d^	R^2,e^	*P* ^d^	R^2,e^	*P* ^d^	R^2,e^	*P* ^d^	R^2,e^	*P* ^d^	R^2,e^
FDR	*rp1.1*	PZE-101149049	1.06	192,154,274	n.s.	–	n.s.	–	n.s.	–	**1.62 × 10** ^**− 05**^	**7.6**	3.46 × 10^− 02^	2.3	n.s.	–
		PZE-101149055	1.06	192,158,701	n.s.	–	n.s.	–	n.s.	–	**2.44 × 10** ^**− 05**^	**7.4**	3.28 × 10^− 02^	2.4	n.s.	–
		PZE-101149110	1.06	192,280,029	n.s.	–	n.s.	–	n.s.	–	**1.64 × 10** ^**− 05**^	**7.6**	3.58 × 10^− 02^	2.3	n.s.	–
	*rp3.1*	PZE-103064079	3.04	97,422,248	2.04 × 10^− 04^	4.72	5.13 × 10^− 03^	2.8	6.66 × 10^− 03^	2.5	3.11 × 10–03	3	**2.94 × 10** ^**− 05**^	**6.4**	8.81x 10^-03^	2.34
	*rp3.2*	PZE-103072633	3.04	115,864,889	n.s.	–	n.s.	–	n.s.	–	n.s.	–	**9.10 × 10** ^**− 06**^	**8.2**	1.86x 10^-02^	1.85
	*rp6.1*	PZE-106060721	6.04	111,526,964	n.s.	–	n.s.	–	**2.54 × 10** ^**−05**^	**7**	n.s.	–	n.s.	–	n.s.	–
	*rp8.2*	PZE-108085787	8.05	141,439,732	n.s.	–	n.s.	–	n.s.	–	n.s.	–	**1.49 × 10** ^**− 05**^	**6.7**	n.s.	–
MDR	*rp1.1*	PZE-101149049	1.06	192,154,274	n.s.	–	n.s.	–	∕	∕	**1.92 × 10** ^**−05**^	**7.7**	n.s.	–	n.s.	–
		PZE-101149110	1.06	192,280,029	n.s.	–	n.s.	–	∕	∕	**1.89 × 10** ^**−05**^	**7.7**	n.s.	–	n.s.	–
	*rp3.1*	PZE-103063977	3.04	97,261,544	2.19 × 10^−04^	4.83	9.30 × 10^−03^	2.51	∕	∕	2.97 × 10^−03^	3.1	**6.77 × 10** ^**−06**^	**7.4**	1.75 x 10^-02^	1.98
		PZE-103063980	3.04	97,261,646	2.19 × 10^− 04^	4.83	9.30 × 10^− 03^	2.51	∕	∕	2.80 × 10^−03^	3.1	**8.34 × 10** ^**− 06**^	**7.3**	1.75 x 10^-02^	1.98
		PZE-103064079	3.04	97,422,248	1.89 × 10^−04^	4.99	4.92 × 10^− 03^	3	∕	∕	2.31 × 10^−03^	3.3	**5.65 × 10** ^**−06**^	**7.7**	8.70^-03^	2.46
		PZA-002742001	3.04	97,441,784	1.26 × 10^−03^	3.74	n.s.	–	∕	∕	1.90 × 10^−02^	1.9	**1.61 × 10** ^**−05**^	**6.8**	4.09 x 10^-02^	1.50
	*rp8.2*	PZE-108085787	8.05	141,439,732	2.30 × 10^− 02^	1.78	n.s.	–	∕	∕	n.s.	–	**9.03 × 10** ^**−06**^	**7.1**	n.s.	–
AUDPC	*rp1.1*	PZE-101149049	1.06	192,154,274	n.s.	–	n.s.	–	∕	∕	**1.07 × 10** ^**−05**^	**8.2**	4.05 × 10^− 02^	2.3	n.s.	–
		PZE-101149055	1.06	192,158,701	n.s.	–	n.s.	–	∕	∕	**2.19 × 10** ^**−05**^	**7.7**	3.03 × 10^− 02^	2.5	n.s.	–
		PZE-101149110	1.06	192,280,029	n.s.	–	n.s.	–	∕	∕	**1.06 × 10** ^**−05**^	**8.2**	4.17 × 10^− 02^	2.2	n.s.	–
	*rp3.1*	PZE-103063942	3.04	97,157,105	2.71 × 10^−04^	5.79	n.s.	–	∕	∕	1.40 × 10^−02^	3	**3.18 × 10** ^**−05**^	**7.6**	5.19 x 10^-03^	2.59
		PZE-103063977	3.04	97,261,544	1.33 × 10^−04^	5.15	2.09 × 10^− 02^	1.85	∕	∕	6.84 × 10^−03^	2.6	**8.42 × 10** ^**−06**^	**7.2**	1.33 x 10^-02^	2.02
		PZE-103063980	3.04	97,261,646	1.33 × 10^−04^	5.15	2.09 × 10^− 02^	1.85	∕	∕	6.63 × 10^−03^	2.6	**1.03 × 10** ^**−05**^	**7.1**	1.33 x 10^-02^	2.02
		PZE-103064079	3.04	97,422,248	1.59 × 10^−04^	5.11	1.27 × 10^− 02^	2.19	∕	∕	5.70 × 10^−03^	2.7	**8.08 × 10** ^**−06**^	**7.4**	8.91 x 10^-03^	2.29
		PZA-002742001	3.04	97,441,784	4.94 × 10^−04^	4.38	n.s.	–	∕	∕	3.24 × 10^−02^	1.6	**1.53 × 10** ^**−05**^	**6.9**	4.38 x 10^-02^	1.37
	*rp5.1*	PZB00182.1	5.02	10,055,423	**2.66 × 10** ^**−05**^	**7.38**	n.s.	–	∕	∕	n.s.	–	1.22 × 10^−02^	3.1	n.s.	–
	*rp8.1*	SYN30855	8.03	72,047,084	**2.33 × 10** ^**−08**^	**12.7**	2.42 × 10^−02^	–	∕	∕	n.s.	–	n.s.	–	n.s.	–
		PZE-108044485	8.03	72,168,101	**1.77 × 10** ^**−05**^	**7.93**	n.s.	–	∕	∕	n.s.	–	n.s.	–	n.s.	–
		PZE-108044552	8.03	72,223,299	**1.69 × 10** ^**−05**^	**7.72**	n.s.	–	∕	∕	n.s.	–	6.70 × 10^−03^	3.5	n.s.	–
		PZE-108044562	8.03	72,238,277	**2.16 × 10** ^**−08**^	**12.7**	2.13 × 10^−02^	2.6	∕	∕	n.s.	–	6.24 × 10^−04^	5.2	n.s.	–
		PZE-108045789	8.03	74,430,017	**1.98 × 10** ^**−08**^	**13**	2.49 × 10^− 02^	2.55	∕	∕	n.s.		2.12 × 10^−04^	6.1	n.s.	–
		PZE-108045901	8.03	74,476,703	**2.22 × 10** ^**−08**^	**12.7**	2.25 × 10^−02^	2.56	∕	∕	n.s.	–	2.10 × 10^− 02^	2.7	n.s.	–
		PZE-108047302	8.03	78,171,783	**1.80 × 10** ^**−05**^	**7.7**	n.s.	–	∕	∕	n.s.	–	n.s.	–	n.s.	–
		PZE-108047365	8.03	78,293,010	**1.47 × 10** ^**−05**^	**7.82**	n.s.	–	∕	∕	n.s.	–	5.32 × 10^−03^	3.7	n.s.	–
		PZE-108047455	8.03	78,555,578	**1.57 × 10** ^**−05**^	**7.79**	n.s.	–	∕	∕	n.s.	–	6.78 × 10^−03^	3.6	n.s.	–
		PZE-108047486	8.03	78,677,089	**1.63 × 10** ^**−05**^	**7.99**	n.s.	–	∕	∕	n.s.	–	6.46 × 10^−03^	3.6	n.s.	–
		PZE-108047488	8.03	78,684,842	**1.74 × 10** ^**−05**^	**7.71**	n.s.	–	∕	∕	n.s.	–	5.53 × 10^−03^	3.7	n.s.	–
		PZE-108047536	8.03	78,758,640	**1.46 × 10** ^**−05**^	**7.83**	n.s.	–	∕	∕	n.s.	–	5.29 × 10^−03^	3.7	n.s.	–
	*rp8.2*	PZE-108085787	8.05	141,439,732	1.95 × 10^−02^	1.86	6.30 × 10^−02^	1.16	∕	∕	n.s.	–	**1.52 × 10** ^**−05**^	**6.7**	n.s.	–
	*rp10.1*	PZE-110093359	10.1	140,987,405	**1.89 × 10** ^**−05**^	**8.78**	2.57 × 10^−02^	2.73	∕	∕	n.s.	–	n.s.	–	n.s.	–

^a^*FDR* final disease rating, *MDR* the mean of disease rating, *AUDPC* the area under disease progress curve

^b^Significant polymorphic sites for each SNP are shown in bold (*P* < 1.07 × 10^−05^ for 32,051 SNPs)

^c^The numbers of the line observed. ^d^*P* value from association analysis carried out using the MLM incorporating population structure and kinship, using the integrated data from 5 locations. *R*^2^ values showing percentage phenotypic variation explained

n.s. indicates not significant at α = 0.05

Based on the genomic region and size with significant SNPs, we classified these SNPs into 8 QTLs (Table 5). Five QTLs associated with FDR were detected, including one QTL denoted as *rp6.1* (Bin 6.04 Pos 111 M) at Embu (Kenya) in 2009, one QTL denoted *rp1.1* (Bin 1.06 Pos 192 M) at El Batan (Mexico) in 2010 and three QTLs denoted as *rp3.1* (Bin 3.04 Pos 97 M), *rp3.2* (Bin 3.04 Pos 115 M) and *rp8.2* (Bin 8.05 Pos 141 M) at El Batan in 2011, respectively. Three QTLs associated with MDR were detected, including one QTL denoted as *rp1.1* (Bin 1.06 Pos 192 M) at El Batan in 2010 and two QTLs denoted as *rp3.1* (Bin 3.04 Pos 97 M) and *rp8.2* (Bin 8.05 Pos 141 M) at El Batan in 2011, respectively. Six QTLs associated with AUDPC were detected, including three QTLs denoted as *rp5.1* (Bin 5.02 Pos 10 M), *rp8.1* (Bin 8.03 Pos 72-78 M) and *rp10.1* (Bin 10.06 Pos 140 M) at El Batan in 2009, one QTL denoted as *rp1.1* (Bin 1.06 Pos 192 M) at El Batan in 2010 and two QTLs denoting as *rp3.1* (Bin 3.04 Pos 97 M) and *rp8.2* (Bin 8.05 Pos 141 M) at El Batan in 2011, respectively.

There were three QTLs associated with all three disease parameters (FDR, MDR and AUDPC) which were located on Chr.1 (*rp1.1*), Chr.3 (*rp3.1*) and Chr.8 (*rp8.2*). All the QTLs associated with MDR were detected for AUDPC as well. One QTL (*rp8.1*) on Chr.8 associated with AUDPC was detected with several significant SNPs with high percentage of PVE > 10%. It is notable that a significant QTL, *rp3.1*, detected for FDR, MDR and AUDPC at El Batan in 2011, was also detected at El Batan in 2009A, 2009B and 2010 with a low *P* value, suggesting that *rp3.1* is likely to be a major QTL.

### Candidate genes annotation of associated SNPs

Candidate genes were selected around the associated SNP (within ~ 200 kb) based on known involvement as metabolic or signaling genes in disease resistance. The gene annotation information was used to identify the putative function of genes around associated SNPs. Nine candidate genes were identified in the significant SNP sites (or adjacent to these sites) of six associated loci (Table 6). The combined approach was not effective for all loci because of the complexity of candidate gene identification. There were several association signals located in genomic regions with tandemly repeated genes. We identified nine candidate gene on chromosomes 1, 5, 6, 8 and 10. Chromosome 5 had two candidate genes (GRMZM2G181002 at 10,084,848–10,087,159 bp, and GRMZM5G829476 at 10,117,318–10,118,871 bp) while chromosome 8 had four candidate genes (Table 6).

**Table 6:** A subset of associated loci and candidate genes identified for common rust resistance according to gene annotation

Chr.	Position (bp)^a^	Candidate genes	Description
1	192,154,274	AC197246.3_FG001^b^	Ras-related protein ARA-4, small GTPase mediated signal transduction
5	10,055,423	GRMZM2G181002^b^, GRMZM5G829476^b^	Phosphotransferases. Serine or threonine-specific kinase subfamily
6	111,526,964	GRMZM2G156712^b^	FMN binding, kinase-associated protein, essential for defense against pathogens
8	72,047,084	GRMZM2G157156^b^	PDZ/DHR/GLGF. Serine signalling proteases with PDZ domains
	78,171,783	GRMZM2G350684^b^, GRMZM2G018048^b^	SANT_DNA-bd. Novel transcriptional regulatory proteins that were identified based on homology to the DNA binding domain of c-myb.
		GRMZM2G018142^b^	HAS protein. Found in helicases and associated with SANT domains
10	140,987,405	GRMZM2G109753^b^	Scramblase protein, responsible for the translocation of phospholipids between the two monolayers of a lipid bilayer of a cell membrane

^a^Position in bp according to B73Ref_V2

^b^These genes have known involvement as metabolic or signaling genes in the disease resistance

### QTL mapping for common rust

The bi-parental RIL population was evaluated for common rust resistance in three environments. Significant phenotypic variation for rust resistance was observed among the RILs (Additional file 5). The genotypic variance (σ^2^_G_) was significant (*P* < 0.01) at single environments. For combined ANOVA σ^2^_GE_ was significant (*P* < 0.01), suggesting common rust resistance is affected by environmental factors. Broad-sense heritability was 0.72 across environments (Additional file 5), revealing that rust resistance was controlled by genetic factors and the data could confidently be used for QTL mapping.

Five QTL were detected in the RIL population, one each on Chr. 1 and 4, and three on Chr. 5 (Table 7). The QTL on Chr.5 (*qRps5–1*) had the highest LOD value (7.74) and it accounted for 18.37% of the total phenotypic variation observed for common rust resistance in the RIL population. The other two QTLs on Chr. 5 (*qRps5–2 and qRps5–3*) explained 15.84% of the phenotypic variation. Combined, the five QTLs detected in the RIL population explained 39.6% of the total phenotypic variance for common rust resistance.

**Table 7:** Estimated quantitative trait loci (QTL) locations and genetic effects affecting common rust resistance in the CML444 × MALAWI RIL population

QTL	Chr.	Position (cM)	Left Marker	Right Marker	LOD	PVE (%)	Add
*qRps1–1*	1	105	PZA03742.1	PHM12323.17	2.87	8.70	−0.15
*qRps4–1*	4	28	PHM3963.33	umc1294	2.62	8.27	0.14
*qRps5–1*	5	118	PZA02207.1	PHM2769.43	7.74	18.37	−0.22
*qRps5–2*	5	140	PZA01349.2	PZA01303.1	4.09	9.90	−0.16
*qRps5–3*	5	193	umc48b	npi237	2.65	5.94	−0.12

*Chr*. Chromosome, *Add* additive effect, *PVE* Phenotypic variation explained

Disease parameter used for QTL analysis was average common rust score

## Discussion

Genetic resistance to maize foliar diseases is the most important, economical and sustainable strategy for managing disease epidemics to increase maize production, especially for smallholder farmers. Development of open pollinated or synthetic maize varieties and hybrids resistant to major diseases requires sufficient information on the genetics and organization of resistance genes on the maize chromosome. This information will allow efficient strategies to combine or pyramid these genes in maize inbred lines that should allow resistant hybrid development. Genome-wide association studies that utilize diverse sets of inbred lines provide an avenue to precisely localize QTLs for quantitative traits and to potentially identify candidate genes [8]. This study used a combination of multiple environment phenotyping of a common set of inbred lines and association mapping to elucidate the genetics of maize resistance to common rust. Results from this study revealed relatively large repeatability estimates for response to common rust at single and across environments. This suggested that actual heritability estimates for common rust may be high, leading to higher genetic gain during selection for resistance to common rust. Higher repeatability estimates may also be attributed to the large diversity of the germplasm used.

Disease parameters, FDR and AUDPC are among those used to identify partial resistance to common rust in maize. Bailey et a1. [31] suggested the use of AUDPC to identify partial resistance to plant diseases for different crops, as this is an integrative parameter that measures the rate of disease progress as opposed to the final disease ratings. Hence, AUDPC can be useful in the identification of QTL that are associated with different components of disease resistance. Although a very strong correlation was observed between FDR and AUDPC (*r* = 0.97), these two parameters could be associated with different types of resistance. Three QTL, *rp1* on Chr.1, *rp3.1* on Chr.3 and *rp8.2* on Chr.8, were detected by all three (FDR, MDR and AUDPC) disease parameters. All the QTL associated with MDR were detected with AUDPC. More SNPs were detected for AUDPC than for FDR, further indicating the importance of using different parameters in association mapping. Although it costs more (time and labor) to obtain data to calculate AUDPC because several ratings must be performed during crop development/growth cycle, our study has shown that it is more effective than a single score for QTL discovery.

Association analysis revealed common rust resistance QTLs on chromosomes 1, 3, 5, 6, 8 and 10, and these are in the regions that have previously been reported to harbor *P. sorghi* resistance [7]. Some of the QTL identified in this study have been mapped to regions previously described to be associated with common rust resistance through bi-parental population–based linkage analysis [3, 6, 24] and other methods of analysis [5, 8, 32–34]. Lübberstedt et al. [3] reported that linkage groups 1 (bin1:05–1:06), 6 (6:04), and 10 (10:05–06) harbored important QTL for common rust resistance. In these regions, we also detected significant associations through GWAS, meaning that the action of these polymorphism loci may be influenced by linked QTL on the same chromosome. Brown et al. [24] identified QTL in bins 2.05 and 5.02 that confer partial resistance to common rust in maize. These bins correspond to association locations identified in our study. Two QTLs identified in this study (in bins 3.04 and 8.03) were also identified by Olukolu et al. [8]. This suggested the need to initiate a fine mapping study for common rust by targeting the common regions identified by various research groups with diverse germplasm. Furthermore, some association loci (*rp8.1, rp8.2*, *rp10.1)* that confer partial resistance to common rust have not been previously reported. Chromosome 10 has been reported to harbor genes for resistance to southern corn rust [35] but we do not have information if it is the same or different set of genes as those for common rust. In our study, the QTL, *rp3.1*, detected using all three common rust resistance parameters (FDR, MDR and AUDPC) at El Batan in 2011, was also found at El Batan in 2009A, 2009B, and 2010 although with a non-significant low *P* value. This suggests that *rp3.1* may be a major QTL associated with resistance against common rust and it warrants further investigation.

Sources of quantitative disease resistance in crop plants have proven to be highly durable [36], making it a promising breeding target for long-term common rust resistance. The integration of resistance into adapted maize germplasm is, however, difficult because it is multi-genic, thereby making backcrossing inefficient. Difficulties in phenotyping common rust further complicate the breeding efforts. As with other diseases, breeding for common rust resistance requires artificial inoculation for uniform pathogen pressure to identify susceptible and resistant genotypes with little chance of escapes. In nature, the infrequent occurrence of the maize rust pathogen has resulted in inconsistent selection between environments, which has led to difficulties in selecting for and maintaining common rust resistance in maize breeding lines [37]. In the absence of selection pressure, resistance alleles may be lost, especially those with minor effects on resistance, as has occurred before [38]. In our study, no QTL was common across locations when using AUDPC, suggesting high pathogen variation among the locations. In this case, it might be more effective to use marker-assisted selection for loci linked to major and partial-resistance QTL to develop common rust resistant inbred lines and hybrids. Marker assisted selection has been successfully deployed for traits that are simply inherited, and is justified for such traits that are either too difficult or expensive to phenotype [39].

In this study, flowering time and common rust FDR were negatively correlated. This suggested that reaction to common rust was independent of genotype maturity. This result corroborates findings by Carson et al. [40] for southern leaf blight but is in contrast to Liu et al. [41] for gray leaf spot (GLS). Associated loci for FDR and flowering time did not co-localize (data not shown), a result that is in contrast to findings in other studies with maize diseases [40]. This is surprising since common rust, like other foliar diseases of maize, tends to be a late-season disease and earlier materials tend to escape.

In maize, host plant resistance genes are frequently found in clusters; however, the statistical power of current mapping techniques does not allow for further resolution of whether these genes are contiguous or allelic to known genes. Huang et al. [42] identified candidate genes for 18 associated loci through detailed annotation in rice, thus showing that the integrated approach of sequence-based GWAS and functional genome annotation has the potential to match complex traits to their causal polymorphisms. In our study, we identified candidate genes in the associated loci on chromosomes 1, 5, 6, 8, and 10 based on known involvement as metabolic or signaling genes in the corresponding traits. The four candidate genes identified on chromosome 8 are different from those reported in temperate germplasm by Olukolu et al. [8]. There were several association signals located in genomic regions with tandemly repeated genes. The candidate genes on chromosome 5 (GRMZM2G181002 and GRMZM5G829476) encode a phosphotransferases of serine or threonine-specific kinase (STK) subfamily, which play a key role in disease resistance system of plants, and were adjacent to associated loci SNP marker PZB00182.1 (Chr. 5 at 10,055,423 bp). Another gene, GRMZM2G156712 encoding a kinase-associated FMN binding protein, which is essential for defense against pathogens, was adjacent to associated loci SNP marker PZE-106060721 (Chr. 6 at 111,526,964 bp). Candidate genes near the significant associated loci detected by GWAS, maybe involved in the common rust resistance defense system in maize. More work is required to elucidate the potential function of these candidate genes.

## Conclusions

We used a diverse set of inbred lines genotyped with high density markers and evaluated for common rust resistance in multiple environments, and identified QTL significantly associated with resistance to common rust and several candidate genes. The results of this study should be used to fine map common rust resistance by targeting the common regions identified between this and other studies that used different germplasm.

## Methods

### Maize germplasm and phenotyping conditions

A collection of 296 tropical maize inbred lines representing some of the genetic diversity available in CIMMYT’s and IITA’s stress breeding programs (drought, low N, acid soils, and biotic stresses) and denoted as Drought Tolerant Maize for Africa (DTMA) panel was used in this study (Table 8). The detail information about each inbred line constituting the panel is presented in Additional file 3.

**Table 8:** Origin, source and grain color of tropical maize inbred lines included in the Drought Tolerant Maize for Africa (DTMA) panel

Type	Breeding program of source germplasm	Number of Lines	Major categories	Grain color	
				White	Yellow
A	Zimbabwe	41	CMLs^a^, CIMCALI, DTPW^b^	41	0
B	Nigeria	4	KU, P43	2	2
C	Ethiopia	2	Pool9	2	0
D	Colombia	27	SA3, SA4, SA5, SA6, SA7, SA8	4	23
E	Mexico highland	5	A.T.Z.T.R.L.BA90	1	4
F	Mexico entomology	48	CMLs, MBR^d^, ZM607, KILIMA, P84	33	15
G	Mexico subtropical	41	CML, MBR, SPMAT, Pop33, Pop45, Pop501, Pop502	25	16
H	Mexico tropical	44	CML, CLQ, CL	23	21
I	Selection under drought	52	DTPW, DTPY^c^, LPS^e^	41	11
J	Selection under low nitrogen	32	DTPW, DTPY, LPS	24	8
Total		296		196	100

^a^*CML* CIMMYT maize line

^b^*DTPW* Drought tolerant population white

^c^*DTPY* Drought tolerant population white

^d^*MBR* multiple borer resistant

^e^*LPS* La Posta Sequia

The inbred lines were evaluated for response to *P. sorghi* in field trials in six environments in two countries. Field trials were planted in 2009, 2010 and 2011 in Mexico and in 2009 in Kenya (Table 9). Lines were planted in 2 m single-row plots, 0.75 m between rows, and 0.20 m within row to give a total of 10 plants per plot. Trails were laid out in an alpha-lattice design with three replications. Trials at El Batan (19°52’ N, 98°84’ W; 2240 masl) in Mexico were artificially inoculated with *P. sorghi* isolates at the six to eight leaf stage. The El Batan experimental location harbors *Oxalis latifolia*, an alternate host of *P. sorghi*, the rust population at this location is complex as sexual reproduction takes place, resulting in new pathotypes, and therefore artificial inoculation was used. Another trial in Mexico at Celaya (20°35’ N, 100°49’ W; 1778 masl) was planted under natural disease pressure. The trial in Kenya was planted at Embu (0°30’S, 37°27′E; 1350 masl) under natural disease pressure. Both Celaya and Embu are maize disease hotspots including common rust among others. The experimental design used was an alpha-lattice [43] with three replications at all locations. At Embu, plot length was a single 3 m row with inter and intra-row spacing of 0.75 m and 0.25 m, respectively. A recombinant inbred line (RIL) population consisting of 234 families developed from the cross CML444 (R) × MALAWI (S) was also used. This RIL population was developed by Global Maize Program of CIMMYT using the single-seed descent method [44]. The RIL population and its two parents were planted for three seasons at El Batan in 2009 (BA09–1, BA09–2) and 2010 (BA10) to evaluate their reaction to common rust.

**Table 9:** Locations, number of inbred lines and year of evaluation, rainfall, and relative humidity during growing season of the DTMA panel for common rust disease

Experimental location	Year	Code	Number of lines	Planting date	Harvest date	Type of inoculation	Rainfall (mm)	Relative humidity
El Batan, Mexico (Site 1)	2009	BM09A	295	16 Apr 2009	27 Oct 2009	Artificial	725	65.9
El Batan, Mexico (Site 2)	2009	BM09B	295	16 Apr 2009	27 Oct 2009	Natural	725	65.9
Embu, Kenya	2009	Kenya09	292	26 Oct 2009	30 Mar 2010	Natural	578	72.2
El Batan, Mexico	2010	BM10	294	8 June 2010	8 Dec 2010	Artificial	790	76.2
El Batan, Mexico	2011	BM11	296	12 May 2011	2 Nov 2011	Artificial	760	71.0
Celaya, Mexico	2012	Celaya12	296	13 June 2012	16 Nov 2012	Natural	475	63.5

### Disease establishment and phenotyping

**Fig. 4:**
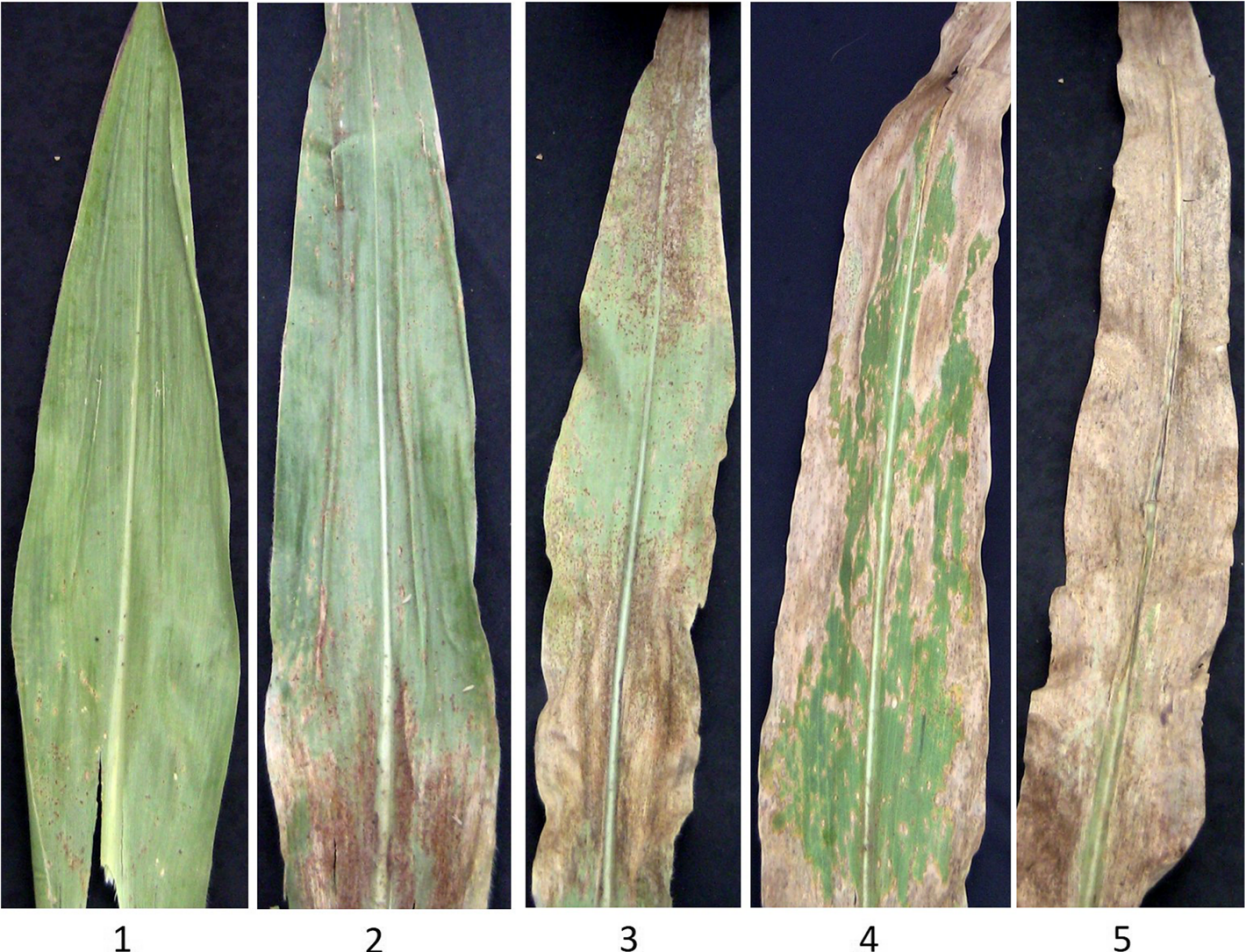
Rating scale used to classify maize inbred lines into disease severity classes. Disease was scored on five-point scale based on the percent leaf area affected by pustules where 1 = 0 to 10% of leaf surface diseased, 2 = 10 to 25% of leaf surface diseased, 3 = 25 to 50% of leaf surface diseased, 4 = 50 to 75% of leaf surface diseased, and 5 = over 75% of leaf surface diseased

Common rust epidemics were initiated artificially by injecting an aqueous suspension of *P. sorghi* spores (60,000 spores ml^− 1^) prepared by mixing sterile distilled water containing 0.03% Tween 20 into the whorl of maize plants at the 6–8 leaf stage. These procedures followed standard techniques for isolation, incubation, and inoculation for common leaf rust. Disease rating was conducted thrice at 15 day-intervals starting one week after silking at all locations, except Kenya09 where rating was done once at the peak of disease symptom expression. Disease rating was scored on five-point scale based on the percent leaf area affected by pustules and impact of the disease where 1 = 0 to 10% of leaf surface diseased (no rust pustules or a few pustules scattered on the leaf surface), 2 = 10 to 25% of leaf surface diseased (numerous pustules on the leaf surfaces), 3 = 25 to 50% of leaf surface diseased (many pustules over the leaf surfaces), 4 = 50 to 75% of leaf surface diseased (many pustules surrounded with huge blighted and sometimes rusty chlorotic zones), and 5 = over 75% of leaf surface diseased (many huge dry pustules surrounded by dead rusty wilted and blighted areas on the leaves) (Fig. 4). The disease rating data were used to calculate the mean disease rating (MDR) and the area under disease progress curve (AUDPC). Mean disease rating (MDR) was calculated as:

$$ \mathrm{MDR}=\sum \limits_{i=1}^n\left({X}_i\right)/n $$

where *i* = time measures as days after planting when rust rating was conducted and *Xi* = rust rating.

AUDPC was calculated as:

$$ \mathrm{AUDUPC}=\sum \limits_{i=1}^n\left[\left({X}_i+{X}_{i+1}\right)/2\right]\left({T}_{i+1}-{T}_i\right) $$

where *i* = time of rust rating*, Ti* = number of days after inoculation and *X*_*i*_ = rust rating [45]. A third parameter, the final disease rating score (FDR, the third disease rating) was included in the analysis. The MDR, FDR, and AUDPC were used as parameters for statistical analysis and association mapping. Other parameters recorded included days to anthesis (AD) and days to silking (SD), which were used as covariates in GWAS computations, to ascertain whether rust resistance or susceptibility was associated with maturity.

### Statistical analysis of phenotypic data

Phenotypic data from each experiment was analyzed for genotypic effects and genotype–environment interactions using the PROC MIXED command of SAS [46]. As lines were scored three times within a season, best linear unbiased predictions (BLUPs) were calculated from a multivariate mixed model for each rating, and a rust index was calculated by averaging the three BLUPs for each line. Repeatability was estimated for the MDR, FDR and AUDPC in a single location and across environments according to Holland et al. [47]. Pearson correlation coefficient between different phenotypic traits were calculated using the PROC CORR option in SAS [46]. Genotypic correlations (r_g_) between locations were estimated according to Cooper et al. [48] as:

$$ {r}_{g(12)}={r}_{p(12)}/{\left({H}_1^2\times {H}_2^2\right)}^{1/2} $$

in which *r*_*p*(12)_ is the phenotypic correlation between the traits measured in locations 1 and 2, H^2^_1_ and H^2^_2_ are the values of broad-sense heritability for the traits measured in locations 1 and 2, respectively. Cluster analysis using Ward’s minimum variance method [49] was performed to group environments based on genetic correlations among the environments. The SAS commands PROC CLUSTER and PROC TREE were used for cluster analysis and to generate the dendrogram, respectively.

### Single nucleotide polymorphism (SNP) genotyping and genome-wide association analysis

Leaf samples were harvested from 10 plants of each line and bulked for extraction of total genomic DNA. All lines were genotyped using Illummina maize BeadChip with 56,110 SNP markers. Markers with a minor allele frequency (MAF) less than 5% in the lines were excluded from subsequent analyses. For the 56,110 SNPs contained in the chip, 32,051 SNPs were used for association mapping after removing SNPs with low MAF. Population structure and kinship were estimated according to Lu et al. [50]. Population diversity and principal component analysis (PCA) were conducted to visualize the genetic structure, and pairwise relatedness coefficients (kinship matrix) were calculated using TASSEL 3.0 [51]. Neighbor-joining tree and principal component analyses (PCA) were used to infer population structure of the GWAS panel. PCA and genetic relationship matrix were conducted in R software and exactly as described by Mahuku et al. [26]. Genome-wide association analysis was conducted using a mixed linear model (MLM) separately for each environment, as described by Mahuku et al. [26]. The *p* values for each marker were combined using Fisher method as described by Chen [52] and the result used to make a Manhattan plot. The Bonferroni correction threshold [53] was used to obtain the Fisher combined *p* value threshold.

### Candidate gene annotation

To identify candidate genes in loci associated with rust resistance, we used public gene annotation datasets based on a filtered gene set of maize sequence (http://ensembl.gramene.org/Zea_mays/Info/Index). All the annotated genes within ~ 200 kb of significant SNPs were retrieved based on known likely involvement as metabolic or signaling genes in disease resistance. These genes encode proteins containing a central domain with nucleotide binding site (NBS), which binds either ATP or GTP, and carboxy-terminal domain consisting of a series of degenerate leucine-rich repeat residues (LRR) in many crops [54–58].

### Linkage mapping

The RIL population of 234 families from CML444 × MALAWI was genotyped with SNP markers using the KASP (Kompetitive Allele Specific PCR) system by LGC Genomics (https://www.lgcgroup.com) and used for genetic linkage map construction. The “Map” function in software QTL IciMapping [59] was used for linkage analysis. A logarithm-of-odds (LOD) threshold of 3.0 was used to declare linkage between two markers. The SNP marker physical position and “nnTwoOpt” algorithm in IciMapping were used to sequence the marker order. The Kosambi mapping function was used to calculate map distances [60]. The IciMapping method [59] was used for QTL mapping using QTL IciMapping. Scanning interval was set as 1 cM between markers. Missing phenotypes were not used for the QTL analysis. The LOD threshold for QTL detection was set at 2.5. For QTL additive effects, positive and negative signs of the estimates indicated that resistance effects were contributed by MALAWI or CML444, respectively.

## Additional files

Additional file 1: The distribution of the common rust resistance in six environments. (TIFF 935 kb)

Additional file 2: Response of inbred lines constituting the drought tolerant maize for Africa association mapping panel to common rust in different environments. (XLSX 34 kb)

Additional file 3: Pedigree of the tropical maize inbred lines constituting the drought tolerant maize for Africa (DTMA) panel. (XLSX 21 kb)

Additional file 4: Q-Q plots of observed versus expected −log10 (*P* values) plots for common rust in different environments and using three disease evaluation parameters. FDR: Final disease rating; MDR: mean disease rating; and AUDPC: area under disease progress curve. (TIF 75 kb)

Additional file 5: Mean and range of rust resistance in the RIL population together with the variance components and heritability estimates in individual and combined environments. (DOCX 13 kb)

## Abbreviations

AD: days to anthesis
AUDPC: Area under disease progress curve
DTMA: Drought Tolerant Maize for Africa
FDR: final disease rating
GWAS: Genome-wide association studies
MAF: Minor allele frequency
MDR: mean disease rating
MLM: Mixed linear model
QTL: quantitative trait locus (loci)
RIL: Recombinant inbred line
SD: days to silking
SNP: single nucleotide polymorphism
