# Supplementary figures and images for: Combined linkage and association mapping reveal QTL for host plant resistance to common rust (Puccinia sorghi) in tropical maize
Source: BMC Plant Biol. 2018 Nov 29;18:310. doi: 10.1186/s12870-018-1520-1 (PMC6267831; doi:10.1186/s12870-018-1520-1)

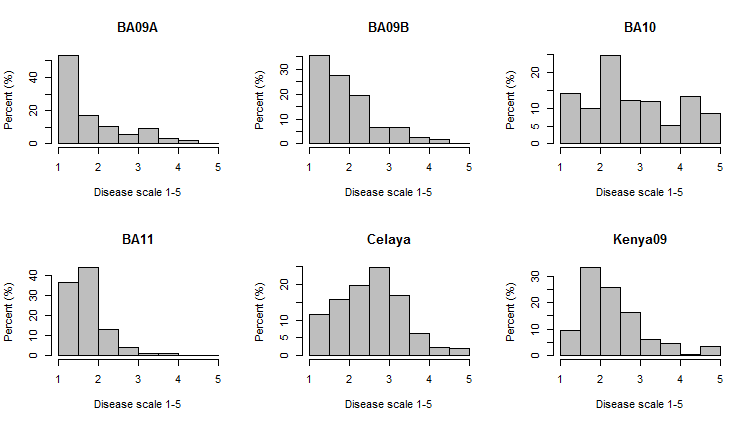

Supplement: Supplementary file 1 — The distribution of the common rust resistance in six environments. (TIFF 935 kb) [file 12870_2018_1520_MOESM1_ESM.tiff]

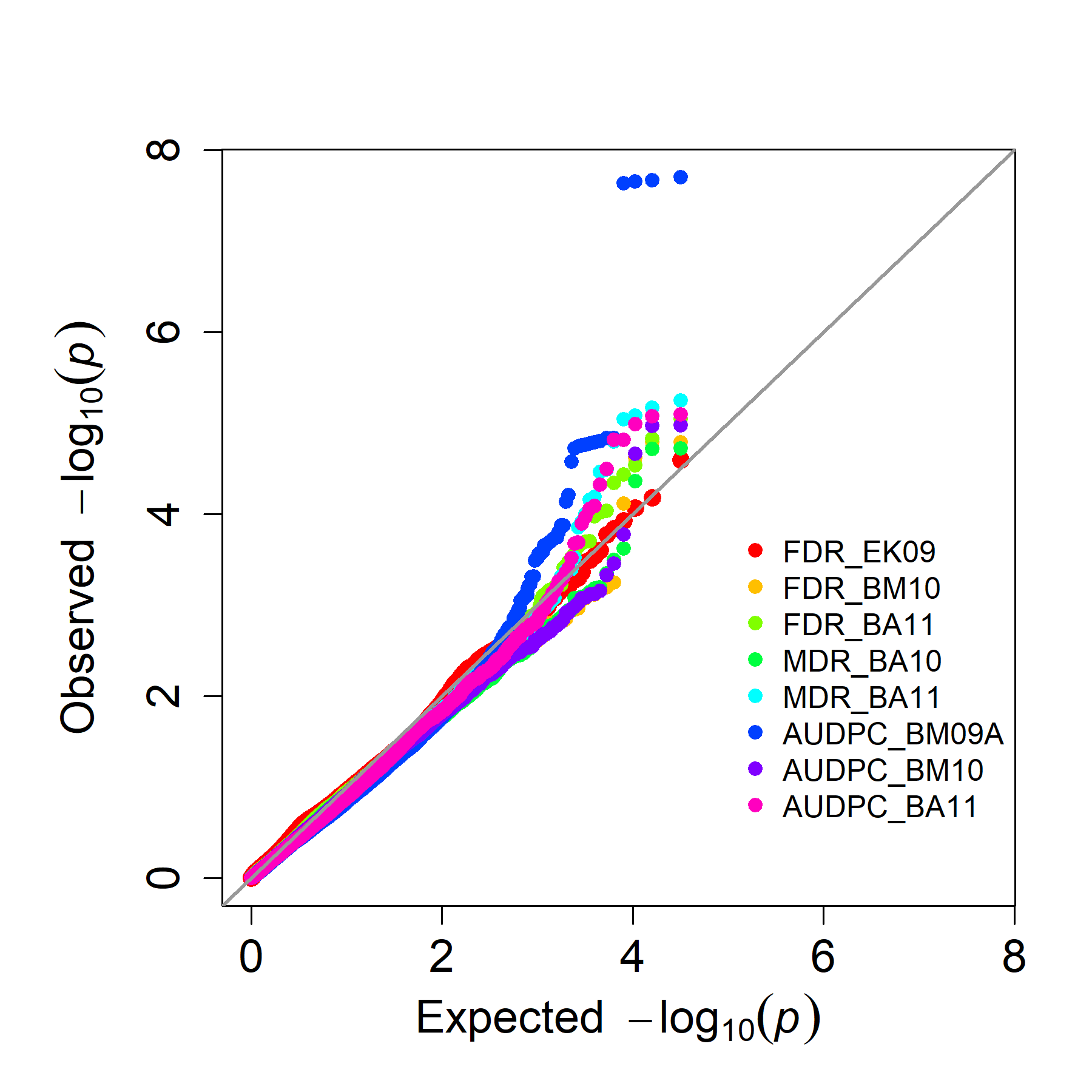

Supplement: Supplementary file 4 — Q-Q plots of observed versus expected −log10 (P values) plots for common rust in different environments and using three disease evaluation parameters. FDR: Final disease rating; MDR: mean disease rating; and AUDPC: area under disease progress curve. (TIF 75 kb) [file 12870_2018_1520_MOESM4_ESM.tif]
