# Supplementary material for: Combined linkage and association mapping reveal QTL for host plant resistance to common rust (Puccinia sorghi) in tropical maize
Source: BMC Plant Biol. 2018 Nov 29;18:310. doi: 10.1186/s12870-018-1520-1 (PMC6267831; doi:10.1186/s12870-018-1520-1)
Supplement: Supplementary file 5 — Mean and range of rust resistance in the RIL population together with the variance components and heritability estimates in individual and combined environments. (DOCX 13 kb) [file 12870_2018_1520_MOESM5_ESM.docx]

**Additional file 5.** Mean and range of common rust disease rating in the CML444 x MALAWI RIL population, genetic ((σ^2^_G_) and genotype x environment ((σ^2^_G_) variance components, and heritability (*h^2^*) estimates for individual and combined environments

|  | | | | | | |
| --- | --- | --- | --- | --- | --- | --- |
| Environments | N | Mean ± SD | Range | σ^2^_G_ ^a^ | σ^2^_GE_ ^b^ | *h^2^* |
| BA09-1 | 220 | 2.23 ± 1.00 | 1-5 | 0.92** | - | 0.84 |
| BA09-2 | 218 | 2.40 ± 0.83 | 1-5 | 0.40** | - | 0.58 |
| BA10 | 200 | 1.71 ± 0.54 | 1-5 | 0.15** |  | 0.53 |
| Combined | 234 | 2.11 ± 0.71 | 1-5 | 0.32** | 0.02** | 0.72 |
|  |  |  |  |  |  |  |

** Significant at the *P* < 0.01 level
